# Risk factors for tissue expander infection in scar reconstruction: a retrospective cohort study of 2374 consecutive cases

**DOI:** 10.1093/burnst/tkaa037

**Published:** 2021-01-04

**Authors:** Chen Dong, Minhui Zhu, Luguang Huang, Wei Liu, Hengxin Liu, Kun Jiang, Zhou Yu, Xianjie Ma

**Affiliations:** 1 Department of Plastic Surgery, Xijing Hospital, Fourth Military Medical University, Xi'an, 710032, Shaanxi, People’s Republic of China; 2 Department of Burn and Plastic Surgery, the Sixth Medical Center of Chinese PLA General Hospital, Beijing, 100048, People’s Republic of China; 3 Information Center, Xijing Hospital, Fourth Military Medical University, Xi'an, 710032, Shaanxi, People’s Republic of China

**Keywords:** Tissue expansion, Tissue expander, Scar reconstruction, Risk factors, Infection

## Abstract

**Background:**

Tissue expansion is used for scar reconstruction owing to its excellent clinical outcomes; however, the complications that emerge from tissue expansion hinder repair. Infection is considered a major complication of tissue expansion. This study aimed to analyze the perioperative risk factors for expander infection.

**Methods:**

A large, retrospective, single-institution observational study was carried out over a 10-year period. The study enrolled consecutive patients who had undergone tissue expansion for scar reconstruction. Demographics, etiological data, expander-related characteristics and postoperative infection were assessed. Univariate and multivariate logistic regression analysis were performed to identify risk factors for expander infection. In addition, we conducted a sensitivity analysis for treatment failure caused by infection as an outcome.

**Results:**

A total of 2374 expanders and 148 cases of expander infection were assessed. Treatment failure caused by infection occurred in 14 expanders. Multivariate logistic regression analysis identified that disease duration of ≤1 year (odds ratio (OR), 2.07; *p* < 0.001), larger volume of expander (200–400 ml vs <200 ml; OR, 1.74; *p* = 0.032; >400 ml vs <200 ml; OR, 1.76; *p* = 0.049), limb location (OR, 2.22; *p* = 0.023) and hematoma evacuation (OR, 2.17; *p* = 0.049) were associated with a high likelihood of expander infection. Disease duration of ≤1 year (OR, 3.88; *p* = 0.015) and hematoma evacuation (OR, 10.35; *p* = 0.001) were so related to high risk of treatment failure.

**Conclusions:**

The rate of expander infection in patients undergoing scar reconstruction was 6.2%. Disease duration of <1 year, expander volume of >200 ml, limb location and postoperative hematoma evacuation were independent risk factors for expander infection.

Highlights

- A large retrospective observational study carried out over a 10-year period including 2374 expanders.
- Disease duration, expander volume, expander site, and haematoma evacuation were related to expander infection.
- Recommendation regarding prevention and management of expander infection according to authors' experience.

## Background

Tissue expansion was first introduced by Neumann in 1957; its widespread clinical application began after Radovan’s representation in 1976 [1, 2]. Scars, which are often reconstructed by tissue expansion, are the sequelae of burn, trauma or surgery [3, 4]. Scars can decrease quality of life for patients and delay reintegration into society due to their perceived appearance causing psychosocial distress [5]. Each year, 100 million patients acquire scars in the developed world, and $12 billion was spent on an anti-scarring drug in the USA alone in 2008 [6, 7]. Although many non-surgical treatments can improve quality of life and the symptoms of scars, none can eliminate scars completely [8]. Given the excellent color and texture match that can be achieved, and the minimal donor-site morbidity of expanded flaps, tissue expansion is often preferred for scar reconstruction [9]. Despite these benefits, a significant hindrance of tissue expansion approaches is the length of time they take and the fact that they require surgery to insert and remove the expander [10]. Moreover, the incidence of complications emerging from tissue expansion varies between 4.0% and 63.0% (mean = 17.4%), according to a systematic review [11]. Once unsalvageable complications occur, the treatment purpose may not be achieved, and new injuries and scars may appear.

Infection is a major threat to the success of tissue expansion; the incidence of infection ranges from 1.4% to 35.4% in previous studies of patients undergoing scar reconstruction [12–18]. The published literature has analyzed possible risk factors for expander infection, including age, gender, body mass index (BMI), prolonged drain use, smoking and radiation therapy, but these studies have presented discrepant conclusions [11, 19–21]. For example, in children, an age of less than 10 years has been shown to be a risk factor, while other studies have found no difference in complication rates between adult and pediatric populations [18, 22]. These discrepancies may be due to small sample sizes, varying definitions of important indicators and differences in surgical methods. Moreover, previous large-sample studies mainly focused on complication risks of expander-based breast reconstruction rather than scar reconstruction [20, 23, 24].

Based on the Clavien–Dindo classification of surgical complications, an infection of Grade II or higher was chosen as the primary outcome in the present study [25]. The cases were considered as expander infection if the following conditions were satisfied: additional antibiotic treatment; unscheduled debridement surgery; or premature removal of tissue expanders because of surgical site infection, but excluding slightly local redness, swelling and bedside drainage. Identifying risk factors for infection can inform potential treatment strategies to reduce the incidence of infection. Thus, we reviewed 2374 consecutive cases of tissue expansion for scar reconstruction over a 10-year period to identify perioperative risk factors for infection from 12 variables.

**Figure 1.**
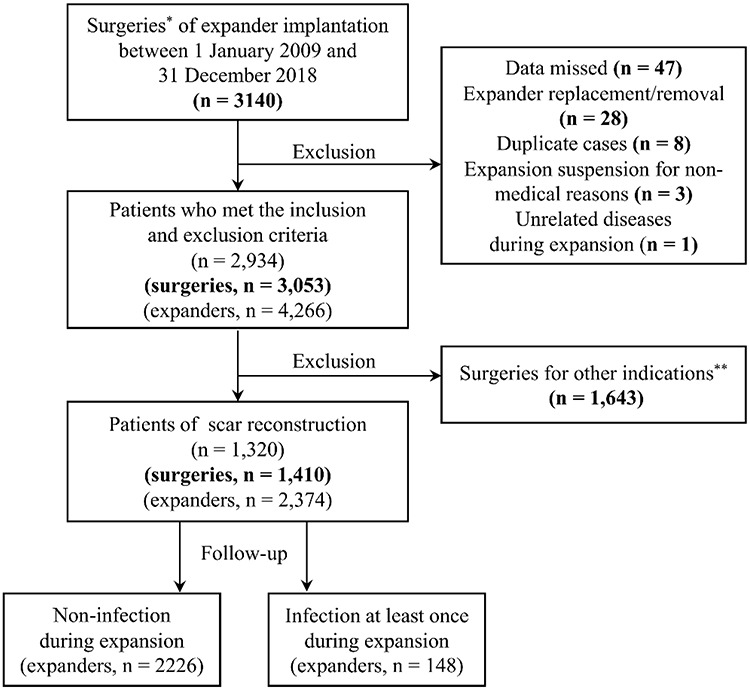
Flow diagram of the 1410 cases (2374 expanders) who underwent tissue expansion for scar reconstruction over a 10-year period. ^*^In one surgery, expander number may be one or more according to practical need. ^**^Other indications include microtia reconstruction, breast reconstruction, penile reconstruction, trauma,etc.

## Methods

### Study design

We identified a retrospective cohort of consecutive patients who underwent expander implantation between 1 January 2009 and 31 December 2018 at a single center (Department of Plastic Surgery, Xijing Hospital). Patients were excluded if they: (1) suffered from unrelated diseases to the extent that normal expansion was disturbed; (2) requested suspension of the expansion for non-medical reasons; or (3) censored key data (e.g. expander volume) for any reason. The study was approved by the ethics committee at Xijing hospital (KY20192155-C-1) and was preregistered in the Chinese Clinical Trial Registry (ChiCTR1900027702).

All variables were collected using the hospital information system (HIS) and extracted using dedicated software (designed by HL and KJ) for tissue expansion. The data were inspected by two independent researchers. Predictors included age, gender, causes, disease duration, preoperative red blood cell (RBC) count, preoperative white blood cell (WBC) count, surgical duration, expander number, expander size, expander location, initial fill volume ratio (initial fill/actual volume) and postoperative hematoma evacuation. The primary outcome was the occurrence of expander infection (beyond Grade II) at least once at any point until the expanded flaps were elevated. The secondary outcome was treatment failure caused by uncontrollable infection. In this situation, the expander had to be removed and tissue expansion was ended. Risk factors and the complication rates were estimated based on each expander.

### Surgical technique

The preoperative design was based on the areas and locations of the lesions. Incisions were made at 1–2 cm from the side of the scar or at the junction between the scar and the normal skin. The length of the incision was 3–7 cm, and the incision was positioned parallel to the long axis of the expander in most cases. The depth of tissue dissection was uniform and depended on the donor and recipient sites. Expanders were inserted between the galea aponeurotica and the periosteal surface in the scalp; beneath the frontal muscle in the forehead; in the superficial layer of the superficial musculo-aponeurotic system in the cheek; beneath the retroauricular fascia in the posterior auricular; in the superficial or deep side of the platysma in the neck; or beneath the deep fascia in the trunk or limbs. The pocket was 0.5–1.0 cm larger than the periphery of the expanders, enabling it to be fully flattened after insertion. All injection ports were external. Negative-pressure drainage was applied routinely. To prevent expanders from displacement under the incision, subcutaneous tissue was sutured approximately 1 cm from the incision and the skin was sutured in layers. Routine bandaging, with appropriate levels of pressure, was applied postoperatively in each case. Intravenous antibiotics were given for 3–5 days, and the drainage tube was removed on either the second or third day after surgery. The suture was removed on day 8–12 after surgery. The first fluid injection into the expander was administered on the third to fifth day after surgery, then once or twice a week thereafter in the outpatient clinic for several months.

### Statistical analysis

Descriptive data is presented as mean ± standard deviation, median (minimum, maximum) or frequencies and proportions, as appropriate, by surgeries. Differences between the groups with or without infection, in terms of their demographic and clinical characteristics, were estimated based on the expander used. The complication rate was also calculated based on the expander. Univariate analysis was conducted using logistic regression modeling. Univariate significance at the level of *p* < 0.1 was considered for entry into a multivariate logistic regression model. The criterion to remain in the model was *p* < 0.05. Furthermore, for validating the robustness of the model mentioned above, we conducted a sensitivity analysis using logistic regression modeling for treatment failure caused by infection. All statistical analyses were conducted using SPSS (version 25.0, IBM, NY,USA).

**Table 1:** Differences between the groups without or with infection in terms of their demographic and clinical characteristics by expanders

Variable	Tissue expander infection, n(%)		Total, n(%)	*P*
	No (n = 2226)	Yes (n = 148)	n = 2374	
Age, years				
<18	718 (32.3)	55 (37.2)	773 (32.6)	1 (reference)
18–40	1414 (63.5)	84 (56.8)	1498 (63.1)	0.156
≥40	94 (4.2)	9 (6.1)	103 (4.3)	0.553
Gender				
Male	1212 (54.4)	88 (59.5)	1300 (54.8)	1 (reference)
Female	1014 (45.6)	60 (40.5)	1074 (45.2)	0.236
Causes				
Thermal burn	1729 (77.7)	117 (79.1)	1846 (77.8)	1 (reference)
Other^a^	497 (22.3)	31 (20.9)	528 (22.2)	0.696
Disease duration, year				
> 1	1811 (81.4)	103 (69.6)	1914 (80.6)	1 (reference)
≤ 1	415 (18.6)	45 (30.4)	460 (19.4)	0.001
RBC count, × 10^12^/L^b^				
4.5–5.5	2044 (91.8)	133 (89.9)	2177 (91.7)	1 (reference)
<4.5	66 (3.0)	3 (2.0)	69 (2.9)	0.548
>5.5	116 (5.2)	12 (8.1)	128 (5.4)	0.142
WBC count, × 10^9^/L^b^				
4.0–10.0	2038 (91.6)	132 (89.2)	2170 (91.4)	1 (reference)
<4.0	116 (5.2)	11 (7.4)	127 (5.3)	0.245
>10.0	72 (3.2)	5 (3.4)	77 (3.2)	0.882
Surgical duration, min				
<60	528 (23.7)	32 (21.6)	560 (23.6)	1 (reference)
60–130	1141 (51.3)	79 (53.4)	1220 (51.4)	0.538
≥130	557 (25.0)	37 (25.0)	594 (25.0)	0.713
Expander number				
1–2	1566 (70.4)	117 (79.1)	1683 (70.9)	1 (reference)
≥3	660 (29.6)	31 (20.9)	691 (29.1)	0.025
Total expander size, ml				
<200	936 (42.0)	46 (31.1)	982 (41.4)	1 (reference)
200–400	544 (24.4)	40 (27.0)	584 (24.6)	0.071
≥400	746 (33.5)	62 (41.9)	808 (34.0)	0.009
Expander location				
Scalp	774 (34.8)	39 (26.4)	813 (34.2)	1 (reference)
Face	470 (21.1)	27 (18.2)	497 (20.9)	0.610
Neck	179 (8.0)	11 (7.4)	190 (8.0)	0.572
Trunk	677 (30.4)	59 (39.9)	736 (31.0)	0.010
Limbs	126 (5.7)	12 (8.1)	138 (5.9)	0.064
Initial fill volume ratio, %				
10–20	1155 (51.9)	72 (48.6)	1227 (51.7)	1 (reference)
<10	438 (19.7)	36 (24.3)	474 (20.0)	0.192
≥20	633 (28.4)	40 (27.0)	673 (28.3)	0.947
Hematoma evacuation				
No	2161 (97.1)	140 (94.6)	2301 (96.9)	1 (reference)
Yes	65 (2.9)	8 (5.4)	73 (3.1)	0.095

*RBC* red blood cell, *WBC* white bloodcell

^a^Other causes: chemical cauterization, mechanical lesion, iatrogenic injury,etc.

^b^Preoperative laboratory test; only current male adult reference is listed here, all subjects are grouped according to the corresponding reference range of different populations and different times

**Figure 2.**
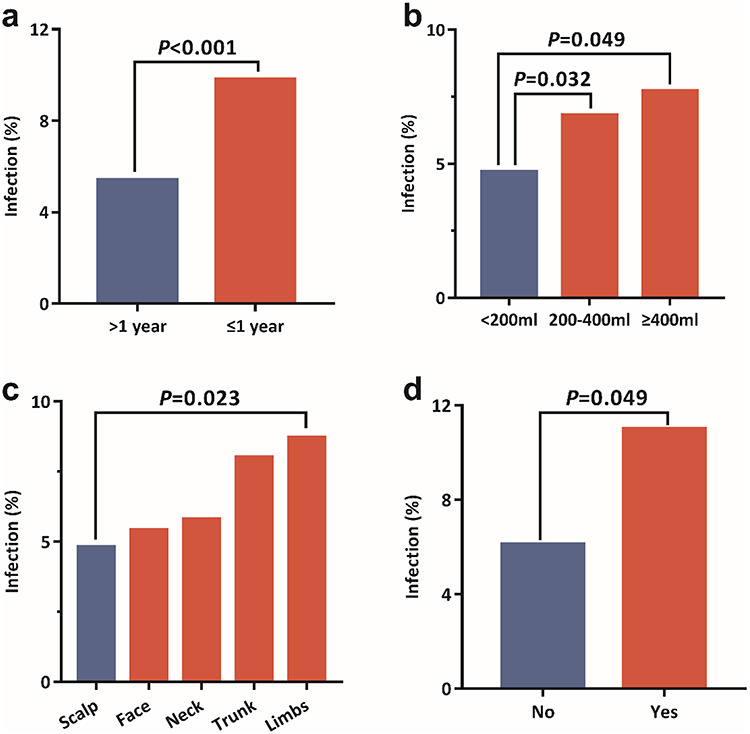
Rate of expander infection in patients undergoing scar reconstruction using expanders according to: **(a)** disease duration; **(b)** expander volume; **(c)** expander site; and **(d)** hematoma evacuation

## Results

A total of 1320 patients and 1410 surgeries were included in the study (Figure 1). The average age of patients was 21.5 ± 9.4 years. A total of 629 (44.6%) patients were female. The main cause of scarring was thermal burns (n = 1017, 72.1%). The length of time from wound healing to the time of surgery was recorded as the disease duration (median = 13.0; range = <1–52 years). Approximately 90% of preoperative laboratory results were within the reference range (RBC, n = 1287, 91.3%; WBC, n = 1279, 90.7%). The median surgical duration of expander insertion was 70 (15, 585) minutes. In 51.5% of cases one expander was inserted (n = 726); 33.9% had two inserted and 14.6% had three or more inserted (n = 478 and 206, respectively). In terms of anatomical locations where expanders were placed, these were: scalp, 31.1% (n = 438); trunk, 28.8% (n = 406); face, 19.5% (n = 275); multiple locations, 12.1% (n = 171); limbs, 4.5% (n = 64); and neck, 4.0% (n = 56). The median total expander size in one surgery was 400 (30, 3350) ml. Additionally, 54 patients (3.8%) required repeat surgery to evacuate a hematoma after expander insertion. The median time of hematoma evacuation was 3 (0, 24) days after the insertion of expanders; there was no expander infection which occurred earlier than the procedures of evacuation.

A total of 2374 expanders were included. One or more infections occurred with 148 expanders (Figure 1). By dividing all data into groups without or with infection and undertaking univariate logistic regression analysis, the differences between the two groups in terms of their demographic and clinical characteristics were calculated and are presented in Table 1.

For univariate logistic regression analysis, five patient features and expanders were selected. The group with disease duration ≤1 year had a significantly higher rate of infection when compared with the group with a disease duration of >1 year (odds ratio (OR), 2.07; 95% CI, 1.42–3.02; *p* < 0.001; 9.8% vs 5.4%, Figure 2a). Expander size was associated with a higher rate of infection—the larger the expander size, the higher the risk of infection (200–400 ml vs <200 ml: OR, 1.74; 95% CI, 1.05–2.90; *p* = 0.032; ≥400 ml, vs <200 ml: OR, 1.76; 95% CI, 1.00–3.07; *p* = 0.049; 7.7% (≥400 ml) vs 6.8% (200–400 ml) vs 4.7% (<200 ml); Figure 2b). Compared with the scalp, expanders placed in the limbs had a higher risk of infection (OR, 2.22; 95% CI, 1.12–4.40; *p* = 0.023; 8.7% vs 4.8%; Figure 2c). The group who underwent hematoma evacuation had a higher rate of infection compared to the normal group (OR, 2.17; 95% CI, 1.00–4.68; *p* = 0.049; 6.1% vs 11.0%; Figure 2d). As for expander number, the difference between 1–2 and ≥3 did not reach statistical significance (*p* = 0.064). Table 2 lists the parameters of the indicative variables in the multivariate logistic regression model and Figure 2 shows the complication rates of the groups mentioned in the model.

**Table 2:** Multivariate logistic regression analysis of risk factors for infection

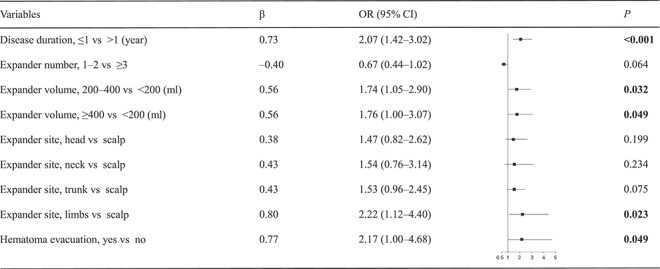

*OR odd ratio, CI* confidence interval

The majority of the infections were controlled timely and had little effect on reconstructive outcome. Unfortunately, 9.5% of expanders (14/148) were permanently removed because of unresolved infection. In the sensitivity analysis for treatment failure caused by infection as outcome, the disease duration of ≤1 year (OR, 3.88; 95% CI, 1.31–11.52; *p* = 0.015) and hematoma evacuation (OR, 10.35; 95% CI, 2.66–40.30; *p* = 0.001) were also related to high risk (Table 3).

## Discussion

Tissue expansion is one of the most important surgical techniques for scar reconstruction, but its relatively high complication rate hinders universal application. Infection, which is one of the major complications of expansion, may result in premature removal and even failure [26]. Therefore, we reviewed 2374 consecutive cases in our clinic to identify risk factors for infection based on hospital admission records.

**Table 3:** Sensitivity analysis for treatment failure caused by infection as outcome

Variables	Univariate		Multivariate	
	OR (95% CI)	*P*	OR (95% CI)	*P*
Disease duration, ≤1 vs >1 (year)	3.15 (1.09–9.12)	0.035	3.88 (1.31–11.52)	0.015
Expander volume, 200–400 vs <200 (ml)	0.84 (0.15–4.60)	0.841	1.39 (0.19–10.29)	0.750
Expander volume, ≥400 vs <200 (ml)	2.45 (0.73–8.15)	0.146	3.68 (0.53–24.96)	0.182
Expander site, head vs scalp	2.46 (0.41–14.79)	0.324	3.31 (0.38–28.65)	0.277
Expander site, neck vs scalp	2.15 (0.19–23.79)	0.534	2.59 (0.21–32.02)	0.457
Expander site, trunk vs scalp	3.89 (0.81–18.80)	0.091	2.52 (0.47–13.62)	0.284
Expander site, limbs vs scalp	2.96 (0.27–32.87)	0.377	4.66 (0.40–54.18)	0.219
Hematoma evacuation, yes vs no	8.92 (2.44–32.69)	0.001	10.35 (2.66–40.30)	0.001

*OR* odds ratio, CI confidence interval

**Table 4:** Algorithm for prevention and management of expander infection

Stage	Measurement
Risk assessment	1. General risks: severe general illness, hemostatic abnormalities, etc.
	2. Primary risks: disease duration of <1 year, hematoma evacuation
	3. Secondary risks: large expander (>400 ml), limb location
Preoperative preparation	1. Proper expander volume and site of expander implantation according to the lesions
	2. Any wound or folliculitis in the donor sites should be noted
	3. Skin cleaning one night before expander implantation
Surgical procedures	1. Strict aseptic technique during surgeries
	2. Thorough hemostasis, endoscope-assisted placement of expander is available [14]
	3. Strenuous and repeated traction of tissue must be avoided
Postoperative care	1. Prophylactic antibiotics (3–5 days)
	2. Any mild infection (e.g. folliculitis) and blood circulation disorders of expanded flap should be treated
	3. Fluid injection by plastic surgery specialized nurses
Infection	1. Oral or intravenous antibiotic regimen (adjusted by culture results)
Mild	1. Remove partial fluid from expander to relax tension of expanded flap
	2. Dressing change
Moderate	1. Expander pocket lavage via drainage tube
	2. Continuous infusion-drainage [40, 41]
Severe	1. Making an incision along the previous one, performing surgical debridement
	2. Expander exchange or removal

The rate of infection was 6.2% of the retrospective cohort during a 10-year period, which was similar to previous studies [11, 27]. The tissue expansion data was characterized by the possibility that one patient may undergo multiple surgeries, and one surgery may include multiple expander insertions, and expanders can be placed at one or multiple anatomical locations. Thus, we described the baseline data by surgeries and calculated the component ratio of the infection groups by expanders. Finally, a disease duration of <1 year, an expander volume of >200 ml, limb location and hematoma evacuation were selected by multivariate regression logistic analysis as independent risk factors for expander infection. To the best of our knowledge, this study included the largest single-center sample of tissue expansion for scar reconstruction to identify risk factors for infection.

The timing of surgical intervention is an important concern for plastic surgeons. Previous practical guidelines for scar management recommend that surgical scar revision may be considered if the patient has developed a permanent scar (existing for at least 1 year) [6, 28, 29]. Our results reveal that a disease duration of <1 year is associated with a higher rate of expander infection. This may be attributed to evidence of inflammation around immature scars, which may affect the anti-infective ability of the expanded local flap [24]. Except in special situations, we recommend 1 year as a reference for the timing of surgical intervention for various types of scar. In addition, it is essential to carefully evaluate the donor area in preoperative preparation.

Expander-related indices, including the number of expanders used per session, the size of the expanders and the location of the expanders were reported as risks for complication of tissue expansion in many articles [10, 11, 21, 22, 30]. Our results confirmed that expanders located in the limbs had the highest infection rate among all body sites and had a significant likelihood of developing infection compared with the scalp. Compared with other anatomical regions, the limbs may have poorer vascular distribution and a larger range of motion. Moreover, the size of the expander, which determined the foreign body size and tissue dissection range, was identified as a significant independent risk factor for expander infection. The OR of infection using the middle-size (200–400 ml) and large (≥400 ml) expanders was 1.74 and 1.77, respectively, when compared with the small (<200 ml) expander. Our findings are in agreement with Karimi *et al*. and Lei *et al*., who recommended that expanders of any size should be expanded to a greater extent instead of choosing a larger expander volume to obtain enough area of expanded flaps [27, 31]. However, our analysis did not show a statistically significant difference in infection rate of based on the expander number per session. In fact, after the skin incision was closed, whether in one or multiple areas, the pocket of each expander was not directly connected. Therefore, in addition to the close proximity, inserting multiple expanders did not significantly increase the possibility of infection with each expander.

Hematoma is an early complication of tissue expansion, usually occurring 0–48 hours after expander insertion. Once uncontrollable postoperative hematoma occurs, reoperations are often required for their removal, which also means hematoma evacuation. In this study, we have shown that hematoma evacuation was associated with a higher likelihood of expander infection. On one hand, tissue hematomas provide a hotbed for bacteria, increase the tension of the expanded flap and prolong the drainage time. Previous studies have shown that hematoma may increase the likelihood of infection of implanted devices or prosthesis [32, 33]. On the other hand, reoperation could increase rate of infection, which has been verified in various kinds of surgeries, such as kidney transplantation, penile prosthesis replacement and shoulder arthroplasty [33–35]. Similarly, surgical re-exploration and evacuation for hematoma were also likely to allow bacteria to enter the pocket of expanders. Therefore, we suggest that the tendency of bleeding should be corrected preoperatively, meticulous care should be taken to achieve intraoperative hemostasis and the duration of prophylactic antibiotics should be extended appropriately for patients who have hematoma evacuation.

In addition, our results suggest that age was not a risk factor for infection, which were consistent with the findings of Adler *et al*, but were not in agreement with other studies [18, 22]. With sufficient preoperative evaluation and meticulous postoperative care, pediatric tissue expansion is safe and effective [26]. Moreover, it has been reported that BMI ≥25, radiation therapy and smoking history can increase the probability of expander complications, but these were not identified in the present study because there were too few patients with a history of radiotherapy or smoking in our study [2, 11, 20, 21, 23]. A partial lack of height data led to invalid analysis of BMI. We also regret having no data to compare infection rate between internal port and external port in this study; this is because, in the past 10 years, all the injection ports of expanders were externally placed in our institution. According to our previous research between 2003 to 2012, the external placement of the injection port did not increase the probability of infection (internal vs external, 5.4% (38/703) vs 5.2% (139/2679)) [36]. Moreover, sterile saline injection via an external port is convenient and painless, especially for the pediatric population [37], and hospitalization is not required during the expansion period (between the insertion and removal of expanders). Given it is a non-invasive operation, saline injection by external port was performed by experienced nurses in the clinic as routine. Therefore, we believe that the external placement of injection port may not be a risk factor for expander infection.

Sensitivity analysis was used to assess the robustness of the research [38]. It is accepted that treatment failure caused by infection is an adverse event for both surgeons and patients. Thus, treatment failure was chosen as another outcome to validate this model. The data in Table 3 indicate that a disease duration of <1 year and hematoma evacuation are also related to a high risk of treatment failure. As for expander volume and site, the difference was not statistically significant, probably owing to the small number (14/2374) of positive patients included.

According to the above analyses, and our clinical experience, we recommend an algorithm for the prevention and management of tissue expander infection, as detailed in Table 4.

The present study adopted a retrospective design. Inherently, the major biases for this study include selection bias and measurement bias [39]. To avoid selection bias, consecutive patients were selected over a 10-year period and the inclusion and exclusion criteria were relatively broad. To avoid measurement bias, the majority of the clinical data were obtained from the HIS by database engineers using special software. These were then checked manually to ensured optimal data accuracy. A small number of patients had missing or inaccurate data; thus, our investigators conducted telephone follow-up interviews with patients to further supplement and revise the correspondingdata.

Despite our efforts to reduce bias, one limitation of this retrospective study was that there was still missing data in the hospital admissions records. Additionally, our research was limited to a single center and should be further verified in a multicenter study. Besides, we intend to gather more cases and reasonably subclassify parts of the body according to whether there are joints or important neurovascular structures nearby for inclusion in future research.

## Conclusions

In conclusion, expander infection in patients undergoing scar reconstruction occurred in 6.2% of cases in our department. A disease duration of <1 year (from wound healing to surgery), an expander volume of >200 ml, expander insertion in the limbs and hematoma evacuation were independent risk factors for expander infection. Disease duration of <1 year and hematoma evacuation were also related to a higher risk of treatment failure caused by infection. We recommend that surgical intervention is performed 1 year after wound healing. When inserting large-volume expanders in the limb region, surgeons should pay extra attention to aseptic technique and postoperative care. Moreover, it is necessary to correct bleeding tendency and properly manage hematoma for infection prevention.

## Data Availability

The data used and/or analysed in the current study are available upon request.

## Abbreviations

BMI: body mass index; HIS: hospital information system; RBC: red blood cell; WBC: white blood cell; OR: odds ratio
